# Supplementary material for: Are carbon emissions trading and green financial instruments synergistic? -Comprehensive quantitative research based on content analysis
Source: PLoS One. 2024 Mar 7;19(3):e0298601. doi: 10.1371/journal.pone.0298601 (PMC10919601; doi:10.1371/journal.pone.0298601)
Supplement: S1 Data — (ZIP) [file pone.0298601.s001.zip › Data/地级市Z_F工作报告.zip!/║┌┴·╜¡/╞δ╞δ╣■╢√╩╨/└┤╫╘╣1⁄2╓┌║┼í╛┬φ┐╦╩2╛▌═°í┐_2010.docx]

各位代表：

　　现在，我代表市人民政府，向大会作政府工作报告，请予审议，并请各位政协委员提出意见。

　　一、2009年工作回顾

　　2009年是极不平凡的一年，是有效应对国际金融危机、保持经济平稳较快发展的一年。我们以科学发展观为指导，在省委、省政府和市委的正确领导下，团结带领广大干部群众，解放思想，真抓实干，全力保增长、保民生、保稳定，圆满完成了市十四届人大三次会议确定的各项任务，国民经济和社会发展取得显著成效。预计完成地区生产总值761.4亿元，同比增长13.8%。财政总收入77.6亿元，为预算的116.5%，增长31%；地方财政收入50.8亿元，为预算的124.6％，增长39.5%；一般预算收入34.5亿元，为预算的119.1％，增长23.5%。财政总支出169.9亿元（包括上级财政补助支出），增长38.1%；地方财政支出107.4亿元，为预算的114.1％，增长24.6％。城镇固定资产投资完成338.2亿元，增长61.8%。谋划和实施重点项目95项，完成投资99.7亿元，其中开工建设67项，有16项完成投资超亿元。争取国家投资项目625项，国家和省投资16.1亿元。2009年是我市项目建设和固定资产投资最多的一年，是我市筹措资金最多、获得资金支持最大的一年，是我市广大人民群众得到实惠最多的一年。

　　（一）农村经济全面发展。大力推进“四个换位”，加快发展现代农业，促进了农业增产、农民增收和农村繁荣。预计农林牧渔业总产值实现321亿元，增长10%。克服严重自然灾害，粮豆薯总产82.85亿公斤，再创历史新高。绿色、有机农产品达到61亿公斤。松嫩平原农业综合开发试验区齐齐哈尔项目区规划建设全面启动。国家航天食品及生物工程研发基地项目已通过国家航天员中心考察论证。现代农业园区、国家有机食品质量监督检验中心和绿色食品检验检测实验中心等项目进展顺利。畜牧业健康发展，预计实现产值120亿元，增长13%。累计转移农村劳动力97万人，实现劳务收入60亿元。农业产业化步伐加快，新上龙头企业15户，完成投资22.8亿元；规模以上龙头企业达178家。县域经济不断壮大，县属工业总产值增长31.7%、利税增长42%，其中飞鹤乳业实缴税金2.2亿元，增长1.5倍。投资14.68亿元，实施17个重点水利项目，中北引扩建、泰来抗旱灌溉引水工程、三大灌区节水改造、城防达标工程和水库除险加固等项目取得突破性进展。投资3154万元，实施黑土区水土流失重点治理工程，新治理水土流失面积13.5万亩。耕地保护工作取得新成绩。强农惠农政策得到落实，发放各类农业补贴资金18.1亿元。争取上级扶贫资金8239.2万元，组织社会帮扶资金1.2亿元，农村贫困人口下降8%。农村现代流通体系进一步完善。卖粮难问题得到较好解决。农村综合改革不断深化，集体林权制度主体改革圆满完成，农村集体经济组织“三资”委托代理服务制全面推行，场县共建取得新进展，户籍管理制度改革深入实施。土地规模经营成效显著，流转土地1125万亩，实现规模经营876万亩。成立农民专业合作组织1780个，其中千万元以上大型农机合作社53个，田间作业综合机械化率达85.6%。新农村建设扎实推进，投入帮建资金1464万元，新增省级示范村、试点村120个。

　　（二）工业经济持续增长。坚持走新型工业化道路，不断加大服务支持企业力度，规模以上工业完成增加值201.2亿元、利税72.3亿元，分别增长17.7%和19%。工业经济效益综合指数预计为195.49，提高14.75点。非公有制经济增加值增长15%。争取国家和省支持企业发展资金1.5亿元。出台了帮助企业保增长促发展的30条措施、工商支持各类经济组织发展的50条措施和更加宽松的困难企业认定标准等政策文件。帮助北钢、齐化、齐轨道交通装备、飞鹤、北玻等企业解决了原料供应、项目建设、产品销售、融资和用地等方面的实际困难，一重、北钢、齐重数控、齐二机床等企业保持了良好的发展态势。完成了8户企业改制，深化了16户企业产权制度改革，化解债务22.1亿元，盘活资产3.8亿元，妥善安置职工5866人。工业大项目建设加快推进，续建和新开工一重铸锻钢基地、传统产业升级改造、大型石化容器及核岛一回路设备国产化，北钢降耗减排，齐重数控高档数控机床产业化，齐二机床高档数控机床产业化、重型数控多工位压力机产业化和腾翔铸锻造基地，齐化“双三十”，飞鹤甘南、龙江、克东乳品加工和润津国际标准化牧场，蒙牛液态奶加工和博天糖业100万吨甜菜加工等28个亿元以上项目，总投资151亿元，完成投资45亿元。已有9个5000万元以上项目竣工投产。国华风电二期工程、安泰生物公司尼森肽有机生物防腐剂产业化、丰源生猪屠宰及深加工、瑞盛食品大豆组织蛋白和隆亿食品獭兔深加工等项目取得积极进展。松花江流域水污染防治规划项目完成投资3.37亿元，4个项目竣工投产。万元GDP综合能耗下降6.6%，削减化学需氧量5253吨、二氧化硫排放量8840吨。

　　（三）第三产业不断壮大。加快发展以旅游业为重点的第三产业，努力促进经济结构优化和产业升级。预计第三产业完成增加值322.1亿元，增长13.9%。实现旅游收入50.1亿元，创汇703万美元，分别增长22.5%和23.6%。高标准完成扎龙5A级景区、尼尔基斯湖景区规划。引资5000万元，启动建设了尼尔基斯湖夏季水上运动项目。扎龙景区和国家森林公园（青松狩猎场）建设取得新进展。与宋城集团签订了投资开发建设昂昂溪俄罗斯风情小镇框架协议。进一步突出关东特色，丰富活动内容，融入中国传统农时节庆活动，使关东文化旅游节成为东北地区重要的旅游会节。着力打造冰雪运动、冰雪文化、冰雪观赏和冰雪观鹤四类项目，冰雪旅游产业发展取得新突破。商贸流通繁荣活跃，汽车、家电等大宗消费持续增长，第九届绿博会、第六届小交会等会展活动成效显著，实现社会消费品零售总额306.8亿元，增长19.2%。完成了综合物流产业园区规划草编。中国马术协会齐齐哈尔马术及速度赛马培训基地项目已获国家有关部门批准，明月岛体育健身中心正式签约落户，大型综合游乐园等项目的前期工作取得积极进展。

　　（四）对外开放深入推进。深入实施大开放、大招商、大经贸战略，大力发展外向型经济。坚持以企业为主体、项目为纽带的务实招商原则，赴香港、澳门、大连等地开展系列招商活动，积极参加哈洽会、津洽会、冬投会等展会，落实招商引资项目701项，其中内资679项，到位资金210.4亿元，增长36.2%；外资22项，到位资金8287万美元，增长25.9%。新引进亿元以上项目31项，总投资90.8亿元，到位资金23.7亿元。对外经贸繁荣活跃，完成进出口总额6.79亿美元，增长16.7%。其中出口4.98亿美元，下降2.3%；进口1.8亿美元，增长1.5倍。哈大齐工业走廊齐齐哈尔建设区取得阶段性成果，新开工10个项目；实现工业总产值80亿元，增长15%。航空口岸通过国家正式验收。南苑高新区争取晋升为国家级高新区取得积极进展。加强对外联系和交流，相继走访和接待了50余家中央部委、70多个国内外大型企业集团和28个国外代表团，与黄冈市缔结友好城市，与大连市签订了经贸合作框架协议。城市知名度和影响力进一步提升，在中国特色魅力城市二百强高峰论坛上，被评为“共和国六十年中国最具投资潜力城市”之一。

　　（五）城乡建设明显加快。坚持以高水准规划引领城乡建设和发展，大力推进城乡重点工程建设，不断完善城乡功能，改善人居环境。高水准完成了哈齐铁路客运专线齐齐哈尔南站、劳动湖水系改造南扩工程、嫩江沿岸发展等规划编制。齐齐哈尔江西新区防洪工程规划已通过水利部松辽委论证。劳动湖水系改造南扩工程全面启动，引水明渠挖掘土方7.8万立方米，南北进出水闸工程顺利开工。橡胶坝工程获得全国水利工程最高奖“大禹奖”。劳动湖音乐喷泉投入使用。投资5221万元，实施卜奎大街、曙光北大街、新江路等12条市政主要街路升级改造，铺装总长度11.7公里、总面积26.9万平方米，分别增长1.2倍和1.1倍。投资1000万元，对劳动湖、龙沙公园、城防江堤及嫩江公园进行了美化亮化。列入省“三供两治”工程的35个项目全部开工建设，完成投资2.5亿元。启动了城区供水管网改造二期工程，改造供、排水管网3788延长米，整合改造泵站75座，接收泵站43座。中心城区热网供热面积达720万平方米，8.5万户居民受益。改造低温楼148栋、71万平方米。投资3.19亿元的压缩天然气加气母站已完成征地、场地封闭等前期工作。在全省率先出台了《商品房预售资金管理办法》，规范了房地产市场秩序。新开发鑫海家园、观湖国际等8个住宅小区，建设商品房106万平方米。切实加强土地宏观调控，摸清土地家底，严把土地闸门，完善征地补偿机制，确保了建设用地需求。哈齐铁路客运专线建设顺利启动，征地拆迁工作全面推进。高速公路建设进展迅速，讷嫩、齐甘公路主体工程正在加快建设，齐泰公路主体工程已交工，齐讷、大齐公路已通车，通车里程165.4公里。中断十年的齐富公路嫩江大桥全面开工，齐泰公路泰来嫩江大桥已经合龙，齐甘公路嫩江大桥已完成部分桩基础。建设农村公路4435公里，新建18个乡镇客运站，在全省率先实现村村通车目标。围绕创建国家级园林城市的目标，加快实施三年造林绿化规划和城区绿化规划，植树造林2.46万公顷，增加1倍以上，在全省造林绿化评比中名列第一；新增城区绿地463公顷，新建阳光文化公园等14个公园、游园，城区绿化覆盖率达到35.4%，提高4.3个百分点，城区绿地率达到34.6%，提高4个百分点，人均公园绿地面积达到8.9平方米，增加1.8平方米。加强城乡环境特别是城乡结合部环境综合整治，加大违章建筑和广告牌匾拆除、墙体立面粉刷力度，市容市貌有了新改观。

　　（六）社会事业全面进步。大力发展各项社会事业，统筹经济社会发展。实施国家重大科技专项11项。新增国家级高新技术企业2户。高新技术产业实现产值288.8亿元、利税30.4亿元，分别增长25.6%和32.2%。1个项目获国家科技进步二等奖，2个项目获省科技进步一等奖，2件发明专利获中国专利优秀奖。高层次人才队伍建设不断加强，10名专家获国务院和省政府特殊津贴。与省科技厅签订科技共商协议并正式实施，成功举办了黑龙江省2009年科技周活动开幕式。与省农科院签署了科技合作共建协议。深入实施中小学提高教育质量行动计划，开展了百所样板校创建活动。扎实推进素质教育，艺体特色学校创建工作取得新成绩。总结推广富裕、克东、泰来等地教育资源整合模式，制定了城市中小学布局调整规划，完成了43所义务教育标准化学校建设任务，促进了城乡教育均衡发展。投资2.7亿元，新建、改建中小学校舍20.3万平方米。落实“两免一补”资金1.9亿元、各级各类助学金3071万元，惠及学生76.4万人次。加强职业教育基础能力建设，中职招生2.1万人，毕业生就业率达95%以上。高考升学率达85.7%。创新方式培训校长和教师1万人次，提高了教育队伍素质。高等教育在校生达4.3万人。投资2770万元，加强了图书馆和文化馆建设。通过市场化运作，对艺术剧院进行了改造。话剧《风刮卜奎》荣获“五个一工程奖”，赴北京、上海、大连等地演出100多场。以兴十四村创业历程为题材，联合拍摄了电视剧《龙头岭》。国家级历史文化名城创建工作扎实开展。原地复建黑龙江将军衙门和将军府项目获得国家文物局充分肯定，正积极推进。古籍藏书楼被国务院命名为全国古籍重点保护单位。6项非物质文化遗产列入国家和省级非物质文化遗产保护名录。成功举办了市第八届体育运动会。在全国第十一届运动会上，我市成为唯一在冬、夏季项目上均获得金牌的城市。获得世界冠军4次、全国冠军47次，运动员王北星获得世锦赛女子大道速度滑冰冠军，白雪在田径世锦赛女子马拉松项目上为国家首夺金牌，冰球队再次夺得全国锦标赛冠军。年内医改任务顺利完成，促进了全市基本医疗保障制度、基本药物制度、基层医疗卫生服务体系、公共卫生服务均等化建设。社区卫生服务人口覆盖率达到90%。甲流防控工作取得阶段性成果。争取资金1100万元，启动了市中医院门急诊病房综合楼建设。我市荣获了省级卫生城市称号。生殖健康促进计划深入实施。人口自然增长率为2.77‰。妇女儿童工作取得新成绩，成功举办了妇女儿童健康暨婚庆文化博览会。扎实开展了“三优两提升”文明城市和法治城市创建活动，市民素质和城市文明程度进一步提高，被评为全国第二届未成年人思想道德建设工作先进城市和国务院授予的全国民族团结进步模范集体。诚信鹤城建设稳步推进，7家企业荣获省诚信示范企业称号。

　　（七）人民生活水平显著提高。着力保障改善民生，提高人民群众生活质量。城镇居民人均可支配收入实现11986.6元，增长8.7%；农村居民人均纯收入实现4637.4元，增长15.03%。居民消费价格总指数上升0.3%，低于控价目标2.7个百分点。深入实施创业带就业工程，出台了为自主创业大中专毕业生提供小额担保贷款、招用就业困难人员享受社保补贴等6个政策性文件。以落实援企稳岗政策为重点，全力稳定就业局势，累计认定困难企业95户，发放三项补贴和降费7702万元，惠及51052名职工。为8900人发放小额贷款2.9亿元，位居全省第一、全国第四。大力开展就业援助活动，继续保持“零就业家庭”动态为零。实现城镇新就业12.4万人，登记失业率为3.72%。社会保障体系不断完善，发放城市低保金3.06亿元、月人均补差95元，发放农村低保金1.02亿元、年人均补差564元，实现了应保尽保。新增城镇职工基本养老保险18860人，32539名“五七工”、“家属工”被纳入基本养老保险统筹范围。在全省率先开展了城乡医保一体化管理，城镇居民参保率达95%，城镇职工参保率达96%，农村常驻人口新农合参合率达95%，将12.98万名困难企业职工纳入了医保范围。全力推进“两改”工程，完成投资15.7亿元，拆迁棚户区123万平方米，建设回迁楼249栋、141万平方米，1.3万户居民迁入新居；完成农村泥草房改造4.7万户、395万平方米，农村住房砖瓦化率提高5.2个百分点。新建廉租住房3834套、15.6万平方米，为39710名“双困”居民发放廉租住房补贴2142万元。为576户农村特困残疾人进行了危房改造。

　　惠民行动”成效显著，发放各类消费券211.7万张，价值5.27亿元，惠及困难群众44.9万户、102万人次。龙沙区获得全国和谐社区建设示范区称号。完成了机关事业单位第二轮规范津贴补贴工作，提高了环卫临时工工资和社区干部生活补贴标准。按照国家有关规定，撤消了境内二级公路收费站。撤消了城区7个路桥收费站，每年免收征费2500多万元。由政府补贴，暂停收取中心城区自来水二次加压费，居民用水每吨减少支出1.2元。加快农村饮水工程建设，解决了10万人饮水安全问题。扶贫救灾工作扎实推进，全市干部群众捐款2532.8万元，发放各类救助资金5881万元，切实保障了困难群众的生产生活。社会治安和安全生产形势保持稳定。加大药品、食品、农资等市场专项整治力度，严厉打击商业欺诈、“霸王条款”、虚假广告等行为，维护了市场经济秩序。认真处理人民群众来信来访，加强领导，落实责任，解决了一批积案和重大信访问题，下大力气解决了原齐纸公司部分人员非正常上访问题。深入开展“五五”普法活动，充分发挥司法行政人民调解、法律服务等职能作用，有效化解了民间纠纷，防止了矛盾激化。积极开展法律援助工作，维护了弱势群体的合法权益。荣获了全国双拥模范城“五连冠”。

　　（八）财税金融工作取得新成绩。全面推进财税增收节支，积极扩大信贷融资规模，努力为经济社会发展提供有力支撑。大力加强财源建设，加大税收征管力度，实现了应收尽收。有9个县（市）区财政总收入突破2亿元，其中龙沙区超过4亿元。落实税收优惠政策，减免税金13.7亿元。加大非税收入征管力度，实现了稳定快速增长。不断深化财政改革，公务卡管理制度全面启动。强化财政监督检查，进一步提高财政资金使用效益。全面实行财政投资评审，有效节约了财政支出。金融业发展步伐加快，成为省金融产品创新和小额贷款公司试点市。各项存款余额772.8亿元，新增133.6亿元，增长20.9%；各项贷款余额476.8亿元，新增135.9亿元，增长39.9%。金融机构存款和贷款的余额存贷比为61.7%，高于全省平均水平6.4个百分点；增量存贷比为101.7%，高于全省平均水平26.2个百分点。金融机构收益8.2亿元，各家银行收益大多在亿元以上。居民人均储蓄由上一年的9300元提高到11300元，人均增加2000元。理顺了担保公司管理体制，商业银行重组圆满完成，依安村镇银行正式营业。企业上市工作进展顺利，一重上市首发申请通过证监会审核。小额贷款公司已批复8家，7家正式营业。与各金融机构签订430亿元贷款授信协议，累计发放到位90.3亿元，占授信总额度的21%。抢抓机遇，积极探索新的融资渠道，首次发行12亿元企业债券获得成功，同时争取省政府转贷地方政府债券3亿元，缓解了建设资金紧张状况。

　　（九）政府自身建设得到加强。自觉接受市人大和市政协监督，依法向市人大报告工作，定期向市政协、市离退休老干部通报情况。人大代表议案和建议、批评、意见以及政协委员提案办结率均达100％。政府法制建设不断强化，依法行政水平进一步提升。制定了市政府重大决策规则、事业单位公开招聘人员暂行办法等制度规定，起草、审修和废止地方性法规（草案）4部、政府规章（草案）7部。56件大事实事基本完成。对39个市直单位的领导干部进行了经济责任审计。查处了23个行政村财经违纪违规问题。对全市领导干部、公务员、行政执法人员进行了分层次的培训。进一步完善了公务用车货币化改革。政府机构改革顺利进行。强化行政效能监督，狠抓政风行风建设，清理自行设立行政审批（许可）事项66项，政府办事效率明显提高。

　　民族宗教、机关事务、新闻出版、统计、档案、地方志、人防、气象、民兵预备役、老龄等工作取得了新成绩。

　　各位代表，过去的一年，我们紧紧依靠市委的领导，坚决落实市委的各项决策部署，抢抓机遇，开拓创新，锐意进取，使各方面工作呈现出欣欣向荣、蒸蒸日上的良好局面，有些工作走在了全国全省前列。前不久，温家宝总理来我市视察，对以齐二机床公司为代表的企业自主创新和以新合家园为代表的棚户区改造工作给予了充分肯定。这是对市委、市政府工作的充分肯定，更是对全市干部群众团结一心、奋发有为、艰苦创业的充分肯定。一年来成绩的取得，是省委、省政府和市委正确领导的结果，是市人大、市政协监督支持的结果，是全市人民共同奋斗的结果。在此，我代表市人民政府，向全市各族人民、各民主党派、各人民团体和各界人士，向所有关心、支持我市改革发展的同志们、朋友们，表示衷心的感谢！

　　在过去一年的工作实践中，我们也积累了宝贵的经验，主要是：

　　——坚持抓超前谋划与抓落实到位相结合，根据宏观经济形势变化和本地实际，积极谋划推进重点领域项目建设，将其作为保增长、保民生、保稳定和结构调整的根本举措，紧抓不放，力促落地，使经济实力和发展后劲明显增强。

　　——坚持抓具体细节与抓问题解决相结合，始终把人民群众的安危冷暖放在心上、抓在手上、体现在实际效果上，努力解决群众特别是困难群众的就学、就业、就医、社保、住房等问题，使全市人民的生产生活条件不断改善。

　　——坚持抓维护绝大多数人的根本利益与抓依法防范惩治极少数人的违法行为相结合，着力解决各类历史积案和遗留问题，使各种影响改革发展稳定的不利因素得到及时化解和妥善处置。

　　——坚持抓争取上级资金支持、扩大招商引资与抓争取信贷支持、发行企业债券相结合，千方百计开辟新的融资渠道，破解资金瓶颈，确保资金安全高效使用和发挥最大效益，为更好更快发展创造必要条件。

　　——坚持抓科学决策、民主决策与抓依法行政、为民执政相结合，要求各级领导干部和广大公务人员，心往一处想，劲往一处使，发扬“5+2”、“白+黑”精神，勤勉敬业，开拓奋进，形成了团结务实、清廉干事、神宁气正的创业氛围。

　　对于这些行之有效的做法，我们在今后工作中，一定继续坚持和完善。

　　我们也清醒地认识到，全市经济社会发展和政府工作中还存在一些困难和问题。在经济发展方面，农业抵御自然灾害和市场风险能力不强，畜牧业比重仍然偏低，绿色食品的品牌建设需要进一步加强，加工规模需要进一步扩大。受国际金融危机影响，部分企业订单减少、增速放缓、效益下滑、出口负增长。对经济增长带动力强的大项目、高新技术项目和国外投资项目不多，受各方面因素制约，有的项目特别是松花江流域水污染防治规划中部分项目进展还不快。市场和流通体系建设需要进一步规范和加强。在社会发展方面，就业压力大，城镇还有3.4万登记失业人员需要安置。市区还有7.5万户、22.2万人住在458万平方米的棚户区里；1.1万户、3.5万人住在136栋、69万平方米的低温楼里。农村还有37.8万户、133.8万人住在2388万平方米的泥草房里。有22.1万平方米的市政道路需要改造。有149个行政村未通硬化公路，有232万农村居民没有吃上自来水。我市人均可支配财力不高，机关公务人员和企事业单位职工收入水平低，特别是还有城镇低收入家庭3.9万户、8.2万人，农村困难户3.8万户、7.2万人。农村教育、文化、卫生事业需要进一步加快发展。群体性矛盾时有发生，大量社会不稳定因素还需要我们着力化解。在政府自身建设方面，少数公务人员的责任意识和工作效能有待提高，不正之风和腐败现象还不同程度地存在。对于这些问题，我们一定高度重视，认真加以解决。

　　二、2010年工作任务2010年是实施“十一五”规划的最后一年，做好今年的政府工作，对于有效应对国际金融危机、保持经济平稳较快发展、为“十二五”规划启动实施奠定良好基础具有十分重要的意义。今年的政府工作，既面临着国内经济形势总体回升向好，国家继续实施积极的财政政策和适度宽松的货币政策、着力提高政策的针对性和灵活性，加大经济结构调整力度，深入实施振兴东北战略等诸多有利条件，也面临着国际金融危机影响仍然存在、经济回升的基础还不牢固等严峻挑战。今年政府工作的总体要求是：坚持以科学发展观为指导，认真贯彻中央和省市委经济工作会议精神，牢牢把握“五个更加注重”，加快落实省建设“八大经济区”、实施“十大工程”的战略部署，按照市委既定的发展定位、发展方向和发展思路，积极应对宏观经济形势变化，全力抢抓发展机遇，以强基础、调结构、增效益、惠民生、促和谐为重点，进一步转变发展方式，加快新型工业化进程，推进新农村建设和第三产业升级，全面完成“十一五”规划目标任务，努力实现经济总量要上新台阶、城乡面貌要有新变化、人民生活要有新提高，促进全市经济社会又好又快、更好更快发展。

　　综合考虑各方面因素，2010年的主要奋斗目标是：

　　——经济总量要上新台阶。过去的十年，是我市经济发展的最好最快时期。国内生产总值增长了两倍，第一产业增加值翻了一番，第二产业增加值翻了两番，第三产业增加值增长了两倍。在这个高起点上，我们要抓住经济回升向好的有利时机，全力推动经济持续快速增长。2010年，地区生产总值同比增长11%，力争13%以上，力争达到850亿元以上；2011年力争突破1000亿元。财政总收入增长13.2%，力争15%以上，达到87.8亿元以上；2011年力争突破100亿元。城镇固定资产投资增长35%以上，力争达到460亿元以上；2011年力争突破600亿元。让我们城市的发展速度更快些，经济增长后劲更强些。加快建设发展动力更加强劲、发展质量更加优良、综合实力更加雄厚的中国绿色食品主产区和集散地、国内外具有重要影响的装备工业基地和全国闻名的生态旅游胜地。

　　——城乡面貌要有新变化。2000年以来，我市坚持高起点规划、高标准建设、高效能管理、高效益经营，大力推进“生态市园林城”建设，使城乡面貌发生了显著变化，获得了中国魅力城市、中国优秀旅游城市、中国最具投资潜力城市等殊荣。我们要立足良好的城乡建设基础，围绕城乡面貌更加整洁、优美、独特的目标，高标准搞好规划建设管理，加快建设国家级园林城市和“三优两提升”文明城市，努力把我市建设成为布局更加合理、功能更加完善、生活更加便利、景观更加优美、文化特征更加突出的北纬47度线以北风格独特的宜居创业型城市。让嫩江水更清澈些，让空气更纯净些，让百姓的屋子更暖些，让群众的出行更便利些，让公园绿地更多些，让大街小巷更加干净些。

　　——人民生活要有新提高。过去的十年，我市城镇居民人均可支配收入和农村居民人均纯收入分别增长了1.7倍和1.3倍，城乡居民生活水平明显改善。我们要更加注重统筹城乡和经济社会发展，2010年城镇居民人均可支配收入增长8%以上，力争达到13000元；2011年力争达到15000元。农村居民人均纯收入增长6%以上，力争达到5000元；2011年力争达到5500元。凡是地方财政担负的各种津贴补贴，都要及时足额兑现；凡需到上级单位争取的，都要努力争取；凡是短期内难以兑现的，都要制定具体可行的中长期规划，认真研究解决办法。让群众的就业机会更多些，经济收入更高些，生活质量更好些。加快建设就业更加充分、保障更加完善、社会更加安定、人民群众更有安全感幸福感的和谐城市和国家级历史文化名城。

　　为实现以上目标，我们要重点做好以下几项工作：

　　（一）加快经济结构战略性调整，全力保持经济平稳较快发展。围绕“经济总量要上新台阶”的目标，强“三农”、壮工业、做大第三产业，力促经济运行质量和效益明显提高。

　　认真抓好“三农”工作。按照国家和省推进农村改革发展稳定的各项部署，深入实施“四个换位”，大力发展现代农业，着力搞好农产品的产加销，加快建设社会主义新农村，促进农村经济全面发展。提高粮食综合产能和品质。加快松嫩平原农业综合开发试验区齐齐哈尔项目区、省千亿斤粮食产能工程、现代农业园区建设。通过调整结构、依靠科技、增加投入等措施，使粮食产能稳定在75亿公斤以上。加大与国家航天员中心的合作力度，力争国家航天食品及生物工程研发基地立项开工，带动绿色食品产业和生物产业发展。推广农业标准化生产，抓好2600万亩优质粮食产业带建设。扩大绿色、特色农作物种植面积，力争达到1000万亩。充分利用国家秋粮收购政策，发挥社会多元主体作用，继续拓宽粮食销售渠道，保证余粮顺畅销售。加强农业基础设施建设。加快三大灌区节水改造、中北引扩建和泰来抗旱灌溉引水工程建设，着力建设一批旱涝保收田和高产稳产田。加快“五区一带”水田开发，新增水田20万亩。继续利用中行贷款，加快千万元现代农机合作社建设。推进农业产业化经营。坚持用现代产业体系提升农业，大力发展食品加工业，扩大加工规模，提升加工层次，培育新的经济增长点。谋划建设年加工300万吨玉米、100万吨大豆、100万吨马铃薯、100万头肉牛、200万头生猪、300万只肉羊、2000万只禽类、300万吨甜菜和300万吨鲜奶等农畜产品精深加工项目。统筹抓好产业龙头、原料基地、农企联结、市场体系四个关键环节，延伸壮大主导产业链条，拉动县域经济加快发展。加快发展畜牧业和劳务经济。优化畜牧业养殖结构，实施良种工程，搞好疫病防治，推进规模养殖，加快建设畜牧业强市，畜牧业产值增长6%，力争10％以上。加强农民职业技能培训，引导农村劳动力有序转移，积极扶持农民工返乡创业就业，力争劳务收入增长16.7%。促进城乡一体化发展。推动资源要素向农村配置，推动城镇化与新农村建设协调发展，推动城乡改革联动，促进城乡统筹协调发展。推进土地适度规模经营，加大政策、资金、信息等扶持力度，力争土地规模经营面积增长14.2%，达到1000万亩。继续深化乡镇机构改革，完善“乡财县管”体制，加大农村集体经济组织“三资”委托代理服务管理力度，壮大村级集体经济。进一步完善集体林权制度配套改革，深化户籍管理制度改革。加快发展农村公共事业，加强农村水、电、路等基础设施建设。坚持开发式扶贫，搞好第五批整村推进，组织全社会力量开展定点帮扶工作，力争使贫困人口下降8%。

　　促进工业结构优化升级。坚持以哈大齐工业走廊齐齐哈尔建设区为牵动，以市场为导向，以科技创新为引领，以大项目为支撑，进一步转变发展方式，加快新型工业化进程。规模以上工业增加值增长12%以上。支持企业自主创新。积极争取国家支持企业自主创新的相关政策，最大程度发挥政策效应。争创国家科技创新试点城市，争取国家数控重型装备产业示范基地申报得到批复。扶持企业创建国家级、省级研发机构，加快科技成果转化。鼓励生物工程、新材料等高新技术产业加快发展，力争高新技术产业产值增长20%以上。支持企业加快研发技改，力争开发新产品65种，实施重点技术改造45项。加大商标品牌战略实施力度，支持企业创品牌、争名牌，进一步提升品牌创建层次和质量。加快大项目建设。重点抓好24个投资超亿元的项目建设，力争完成投资47亿元。引导鼓励支持企业整合资源，运作科技水准更高、经济效益更好、发展前景更大的大项目、好项目。创新大项目建设生成、管理服务和推进落实机制，保障项目建设加快进行。促进企业集团化和集群化发展。支持骨干企业积极参与国内外资源整合与合作经营，扩张发展，做大做强。搭建合作平台，推进骨干企业与配套企业对接，加快优势产业集群化发展。认真抓好节能减排。全力完成松花江流域水污染防治规划项目建设，实现主要污染物减排目标。全面落实节能减排责任制，加强对投资项目的环境评价和重点耗能企业的监管，推广应用节约能源资源的新技术、新设备和新材料。万元GDP综合能耗下降6%以上。进一步加大服务支持企业力度。认真落实国家和省市保增长促发展的政策措施，积极帮助企业解决项目、资金、用地、运输、社保等难题，全力支持企业扩大生产经营。支持非公有制经济发展。完善促进非公有制经济发展的体制机制，着力营造公平竞争的市场环境，进一步放宽市场准入，鼓励民间资本进入基础设施建设、公用事业、金融服务、社会事业等领域。加大对中小企业服务支持力度，努力培育壮大一批信誉优、前景好的民营企业。继续深化国有企业改革。创造条件推进未改制国有企业进行产权制度改革，确保改制一户、成功一户。择机启动厂办大集体改革。抓好企业分离办社会工作，完成市属企办中小学校移交，适当提高“三无”企业退休教师待遇，搞好省属企业分离办社会及铁路尚未移交单位接收工作。加强国有资产监管，实现保值增值。

　　提高第三产业发展水平。以旅游、物流、会展、信息服务外包等产业为重点，加快大项目建设，不断壮大产业规模，提升产业发展水平。促进旅游业加快发展。认真落实国务院和省市政府关于加快发展旅游业的意见，结合省建设北国风光特色旅游开发区和重点旅游城镇，高标准完成全市旅游总体规划和专项规划，着力规划建设一批旅游名镇，打造旅游品牌。拓宽旅游建设融资渠道，积极引进大型旅游开发企业，支持中小旅游企业发展。高水平建设好扎龙、尼尔基斯湖、昂昂溪俄罗斯风情小镇、国家森林公园等主要景区，全力打造“生态旅游区”。搞好明月岛体育健身中心、大型综合游乐园规划建设。培育和发展少数民族文化特色旅游。加大旅游配套设施建设力度，丰富游览内容，延长游览时间，提高接待服务水平。举办好特色更加鲜明、影响更加广泛的关东文化旅游节和冰雪游览会。力争旅游业总收入增长16%以上。培育壮大物流、会展和信息服务外包业。加快综合物流产业园区规划建设，积极引进培育发展物流企业。高质量办好绿博会、小交会，积极承办各类展会。搞好服务外包园区规划建设，积极引进知名IT企业和人才。繁荣商贸流通业。抓好市场体系规划建设，发展新型商贸流通业，促进各类专业批发市场发展。搞好百花园小商品商务中心建设。扎实推进“万村千乡”市场工程，落实好“家电下乡”、“汽车下乡”补贴政策。强化市场监管，规范市场秩序。社会消费品零售总额增长15%以上，居民消费价格总指数涨幅控制在3%左右。

　　进一步扩大对外开放。继续拓展对外开放的广度和深度，大力发展外向型经济。促进外贸稳定增长。抢抓省建设东北亚经济贸易区的有利机遇，大力开展以对俄为重点的经贸合作，继续推进对日、对韩经贸科技合作升级，积极探索与蒙古在资源开采和加工方面合作。引导企业优化进出口产品结构，加大对机电产品、高新技术产品和绿色农产品出口的支持力度，扩大先进技术、关键设备、重要原材料的进口。加快实施“科技兴贸”战略，研究制定扶持政策措施，增强企业出口创汇能力。进出口总额增长10%以上。继续开展务实招商。创新招商方式，组织好以企业为主体、项目为纽带的招商活动。结合资源和产业优势，瞄准国内外500强企业，积极开展定向定位招商推介，着力引进一批大项目。根据国家产业导向和我市比较优势，不断充实完善项目库，谋划包装一批吸引力强的项目。搞好园区和产业基地规划建设，加大投入，完善功能，提高承载孵化项目能力。认真落实招商引资优惠政策，进一步优化审批制度和服务方式。抓好签约项目落实，切实提高资金到位率。扩大对外联系和宣传。进一步加强与知名企业、商会、财团和国内外大中城市的沟通联系，促进政府间、企业间和民间的交流与合作。创新对外宣传方式，提升城市形象。

　　（二）切实搞好城乡规划建设管理，进一步提高城市品位。围绕“城乡面貌要有新变化”的目标，坚持“四高”原则，努力彰显北纬47度线以北的城市独特风格。

　　高标准搞好城乡规划设计。围绕我市具有的平原城市、滨水城市、冰雪城市、多风城市和丹顶鹤之乡等特点，高水准搞好城市规划设计，突出城市总体风格的独特性和亲水性，突出发展空间的开阔性和持续性，突出建筑的艺术性和协调性，突出绿化的集聚性和功能性，突出景观的自然性和人文性，突出交通的便捷性和前瞻性，突出功能的完备性和便民性。充分利用我市独特的资源和条件，精心打造亲水景观、借风景观和特色景观。突出各功能区作用，形成各具特色的城市区域。

　　推进城乡基础设施建设。完善居民生活配套设施，加快城区地下管网改建、热网工程和东南城区热源项目建设，完成卜奎大街南段、曙光大街北段、站前大街北段、庆云街等街路升级改造。积极配合省搞好铁路客运专线南站、高速公路建设。继续推进农村公路建设，完善农村路网。加快劳动湖水系改造南扩工程和嫩江沿岸开发建设，确保完成年内既定任务。推动齐齐哈尔江西新区防洪工程尽快立项实施。抢抓省建设一批工业重镇、商贸大镇的有利机遇，进一步搞好小城镇建设。加大土地收储力度，为城市基础设施建设提供用地保障。

　　高水平规划建设南苑新区。以铁路客运专线南站建设为契机，在南苑规划建设一座现代化新城区。积极争取南苑高新区晋级为国家级高新技术产业开发区。围绕加快装备制造业发展，促进企业技术中心和重点实验室集聚，加快教育科研城建设。围绕加快食品工业发展，搞好国家有机食品质量监督检验中心和绿色食品检验检测实验中心规划建设。围绕第三产业发展，加快中国马术协会齐齐哈尔马术及速度赛马培训基地建设。围绕完善园区功能，加快医院、学校和商贸设施建设。结合滨水城市建设，搞好劳动湖水系改造南扩工程沿岸开发，适时启动铁路客运专线南站环境、基础设施和城市道路建设。结合高速公路建设，加快构建与其相衔接的新区路网。适当调整哈大齐工业走廊齐齐哈尔建设区规划，将原有骨干企业纳入规划范围，新建企业集中向园区摆放。

　　加强生态环境建设。加快实施三年造林绿化规划和城区绿化规划，突出抓好重点绿化工程建设，完成植树造林2.31万公顷、城区绿化172公顷。利用废弃工厂场地树木建设城市公园、游园，完成马恒昌公园等4个公园建设，加快建设清末大草房观光园等6个公园。积极做好申报国家级园林城市工作。搞好水土保持和生态建设，大力实施黑土涵养和中低产田改造工程，加快龙江、甘南、泰来等重点区域的生态环境治理，积极推进讷河、富裕国家级生态示范区创建工作。发展低碳绿色经济，倡导低碳健康生活。积极推进扎龙核心区居民搬迁，保障湿地生态安全。

　　营造整洁优美的市容环境。本着人性化的理念，积极探索城市管理的新办法、新途径，科学搞好城市亮化、美化和净化。理顺城市管理体制，加快建立综合执法队伍。建立健全长效机制，继续加强城乡环境综合整治，科学配备环卫设施，突出抓好城乡结合部和铁路、公路沿线脏乱差问题，巩固城乡环境整治成果。

　　（三）更加注重社会建设，着力保障改善民生。围绕“人民生活要有新提高”的目标，全力做好社会就业、社会保障、社会事业发展和社会稳定工作。

　　积极促进社会就业。以扩大就业、稳定就业、鼓励创业为重点，大力实施创业带就业工程，加大帮扶困难企业力度，强化就业和创业培训，引导支持劳动者灵活就业、自主创业。城镇登记失业率控制在4.3%以内。进一步增加小额担保贷款投放，大力开发公益性岗位，积极开展就业援助活动，帮助困难群体就业再就业，力争城镇新增就业9.8万人，实现下岗失业人员再就业7.5万人，其中困难人员就业1.2万人。坚持面向机关事业单位、面向企业、面向市场、面向农村，积极促进大学生就业。认真做好退役军人安置工作。

　　提高社会保障水平。完善最低生活保障制度，做到应保尽保。扩大城镇职工保险覆盖面，开展好城镇居民医保市级统筹和新型农村社会养老保险试点工作，积极推进城乡医保一体化管理。认真解决10.3万名国有关闭破产企业退休人员参加医疗保险问题。加快发展社会福利和慈善事业，完善老年人保障制度，全面推进居家养老服务，鼓励发展社会养老机构，倾力打造孝亲敬老城市和老年友好城市。加强优抚安置、“五保”供养和扶贫解困工作，力争农村“五保”集中供养率达到50%。做好贫困人口医疗救助和法律援助工作。高度关注并认真解决月人均收入略高于低保标准的困难家庭生活问题。积极探索建立“惠民行动”长效机制，有针对性地解决生活困难群体的衣食住行和就医就学等问题。高度重视自来水水质安全和水价公正合理问题，严厉打击偷水逃费行为。规范完善被征地农民的基本生活保障制度。继续加大对革命老区贫困村的扶持力度。实施妇女儿童发展规划，做好迎接国家终期评估准备工作。

　　加快保障性住房建设。深入落实国家促进房地产市场健康发展的政策措施，规范房地产市场秩序。调整商品房供应结构，适当增加中低价位、中小户型普通商品房和经济适用房供应数量，保障中低收入家庭住房需求。全力加快“两改”工程，拆迁棚户区120万平方米以上，建设回迁楼140万平方米以上，改造农村泥草房3.7万户。搞好廉租住房建设和补贴发放，新建廉租房3055套、12.8万平方米，为22848户发放补贴3084万元。逐步分片改造老式低温住宅及供热设施。

　　提高科教水平。加快科技资源共享平台、重点实验室和企业创新中心建设，抓好重点项目实施，提高自主创新水平。加强科技交流与合作，大力普及科学知识。深入实施人才强市战略，搞好人才培养、引进和使用。深入实施中小学提高教育质量行动计划，推进特色办学，改革中考制度，进一步提高基础教育质量。落实义务教育经费保障政策和助学政策，保障贫困家庭子女就学。加强义务教育标准化学校建设，实施校舍安全工程和农村远程教育工程，加大教育资源整合力度，促进义务教育均衡发展。深化职业教育改革，推进职业教育内涵发展。加强校长和教师队伍培训，提高素质教育能力。重视民族教育、民办教育、学前教育和特殊教育，积极发展高等教育，支持齐齐哈尔职业学院升格为本科院校，促进各类教育协调发展。

　　推动文体事业发展。推进文化体制改革，完善公共文化服务体系，增强文化团体发展活力。加强县（市）图书馆、文化馆、综合文化站和“农家书屋”等文化设施建设。以《风刮卜奎》、《飞翔》、《嫩水鹤翔》等作品为重点，打造一批文艺精品。创新公共文化服务方式，精心办好各类文化活动。发展特色文化产业，做大做强马戏、话剧、影视基地等文化产业，培育壮大创意产业。加强文化市场监管，营造良好的文化产业发展环境。启动原地复建黑龙江将军衙门和将军府项目，做好藏书楼申报国家重点文物保护单位工作，全面推进国家级历史文化名城创建工作。编制体育产业发展规划，积极引进重大体育赛事和特色体育项目。深入开展全民健身运动，完善群众性体育设施，积极备战参与国内外重大体育赛事。认真做好第六次全国人口普查我市的相关工作。

　　增强公共卫生服务保障能力。深入落实国家和省医药卫生体制改革各项部署，加快建立覆盖城乡的基本医疗卫生制度。完善疾病防控体系，全力做好甲流防治工作，提高应对突发公共卫生事件能力。加快农村三级卫生服务网络建设，培养输送技术骨干，提升医疗卫生服务能力。继续扩大新农合覆盖面，确保参合率达到98%。积极推进社区卫生与城镇职工、居民医疗保险和医疗救助相结合，构建新型城市卫生服务体系。推进市中医院门急诊病房综合楼、市第一医院南院等项目建设。稳定低生育水平，提高出生人口素质，积极争创全国人口和计划生育综合改革示范市。

　　全力维护社会和谐稳定。加强安全生产宣传教育，严格落实安全生产责任制，认真抓好生产安全、消防安全和道路交通安全的治理，坚决遏制重特大安全生产事故发生。继续开展食品、药品、农资、日常生活用品质量安全专项整治活动，保障群众健康安全，维护公平有序的市场经济秩序。深入开展“打黑除恶”专项行动，依法严厉打击刑事犯罪，维护社会治安稳定。进一步加强应急管理，搞好检查和演练，提高预防和处置突发事件能力。坚持属地、属事相结合，深入排查化解矛盾纠纷，引导群众依法理性反映诉求，有效预防处置群体性信访事件。完善领导干部定期接访制度，充分发挥信访督查专员作用，建立信访信息综合分析研判机制，及时发现解决苗头性、趋向性问题。下大力气处理历史遗留的重大信访问题，并防止新的问题发生。认真落实信访工作责任制，对重大信访问题处理实施责任追究。进一步做好人民调解工作。实施重大事项社会稳定风险评估工作，从源头上防止和减少矛盾纠纷。积极开展城乡和谐社区创建活动，充分发挥基层群众组织维护稳定的作用。做好第三届社区换届选举工作。认真贯彻党的民族宗教政策，继续推进民族团结进步事业。加强国防教育，深入开展军民共建活动。

　　（四）扎实做好财税金融工作，为经济社会发展提供保障。坚持发展骨干财源，培育新兴财源，强化税收征管和金融支撑，进一步壮大经济综合实力。

　　提高公共财政保障能力。认真研究、大力发展、做大做强县域经济和城区经济，加强支柱产业、骨干企业、优势产品等重点财源建设，培育壮大县（市）区骨干财源，促进市县财政增收。深化土地使用制度改革，加大经营性用地市场化运作力度，对商业、旅游、娱乐和商品住宅等各类经营性用地，必须以招、拍、挂方式出让，确保土地收益。加强非税收入管理，挖掘财源增收潜力。深化财政国库集中支付制度改革，加强预算监管，进一步规范公务支出行为，加大民生重点项目的投入力度。

　　加强税收征管。认真落实国家和省市税收优惠政策，积极涵养税源。强化税源监控和纳税评估，依法加强对重点行业的税收征管，实现应收尽收。加大税源排查力度，深入开展第三产业税源调查，挖掘税收增长潜力。广泛开展诚信纳税活动，严惩偷逃税款行为，营造依法公平纳税环境。

　　强化金融支撑。深入落实支持银行业发展的意见和金融支持地方经济发展的意见，促进金融业与地方经济健康发展。鼓励金融机构加大信贷支持力度，加快落实430亿元贷款授信协议。切实用好已经到位的12亿元企业债券和省政府转贷地方政府的3亿元债券。积极引进外地商业银行设立分支机构。拓宽企业融资渠道，加快安泰生物、华工机床等企业上市步伐。支持信用担保公司做大做强。积极开展金融知识培训，提高各级领导干部和企业管理人员的融资本领。加强社会信用体系建设，构建以政府诚信为主导、以商业诚信和企业诚信为重点、以个人诚信为基础的社会信用体系建设格局。

　　各位代表！要完成繁重的政府工作任务，必须加强政府自身建设，努力建设更加开拓创新、更加勤勉尽责、更加团结务实、更加廉洁高效的人民满意的政府。我们要切实加强学习，准确把握宏观经济形势，跟踪掌握国家政策走向，不断完善发展思路和政策措施，提高驾驭全局的能力和工作水平。始终保持旺盛的干事创业激情，增强事业心责任感，不回避矛盾和困难，在解决事关全局的大事、难事、要事上下功夫，创造性开展工作。加强政府法制建设，严格按照法律、制度、规则和程序办事；强化行政执法监督检查，加强公共服务监管，坚决防止行政不作为和乱作为。积极稳妥地推进政府机构改革，增强政府服务职能；扩大政务服务大厅服务范围，规范服务程序，改进服务质量，提高服务效率和效果；坚持深入实际调查研究，帮助企业协调解决生产经营中的实际困难，真心实意为群众排忧解难。紧紧依靠市委的领导，自觉接受人大、政协和社会各界监督，及时将政府重大决策、部署和事项向社会公开。认真遵守党风廉政建设的各项规定，加大违法违纪案件查处力度，规范财政资金使用、土地出让、政府采购等行为，防止权力滥用，从源头上预防腐败现象滋生。严格实行行政问责制，对有令不行、决策失误、监管不力、处置不当的要问责，对办事拖拉、推诿扯皮、不求进取、平庸无为的也要问责。

　　各位代表！完成全年各项工作任务，实现经济社会发展预期目标，既是政府的重要职责，也是对全市人民的庄严承诺。让我们在市委的领导下，凝心聚力，埋头苦干，为推动全市经济社会又好又快、更好更快发展而努力奋斗！
